# Electrochemotherapy for Recurrence and/or Metastatic Skin Cancers: A Prospective Case Series in Iran

**DOI:** 10.1177/15330338251338635

**Published:** 2025-05-11

**Authors:** Seyed Mojtaba YazdanParast, Sepideh Mansouri, Farshid Rostami Pouria, Navid Manoochehri, Kosar Namakin, Alvand Naserghandi, Seyed Rouhollah Miri, Habibollah Mahmoodzadeh, Omid Nabavian, Shirin Zaresharifi, Mohammad Abdolahad

**Affiliations:** 1 Nano Electronic Center of Excellence, Nano Bio Electronic Devices Lab, School of Electrical and Computer Engineering, University of Tehran, Tehran, Iran; 2 UT and TUMS Cancer Electronics Research Center, 48439 Tehran University of Medical Sciences, Tehran, Iran; 3 Radiation Oncology Research Center (RORC), Cancer Institute, Tehran University of Medical Sciences, Tehran, Iran; 4 Student Research Committee, 556492 Shahid Beheshti University of Medical Sciences, Tehran, Iran; 5 Department of General Surgery, 48439 Tehran University of Medical Sciences, Cancer Institute, Tehran, Iran; 6 Department of Anesthesiology and Intensive Care, 48439 Tehran University of Medical Sciences, Tehran, Iran; 7 Skin Research Center, 556492 Shahid Beheshti University of Medical Sciences, Tehran, Iran

**Keywords:** adverse effects, electrochemotherapy, skin cancer, response rate, electroporation

## Abstract

**Purpose:**

This study aimed to investigate the efficacy of electrochemotherapy on the three common types of skin cancer, including basal cell carcinoma (BCC), cutaneous squamous cell carcinoma (cSCC), and melanoma.

**Methods:**

26 patients with skin cancer were recruited from single cancer treatment centers from 2022 to 2024. Electrochemotherapy (ECT) was performed to treat the cancerous nodules; all nodules in a patient with multiple lesions were treated. However the biggest lesions were always pointed out (according to European Standard Operating Procedures on Electrochemotherapy protocol) and their clinical response and adverse effects were evaluated during the study.

**Results:**

totally, 104 nodules of 26 patients were assessed. Clinical complete response was achieved in 53 lesions, while partial response was observed in 51 lesions after first month of treatment. The most common adverse effect was pain which was in 65% of cases.

**Conclusion:**

BCC shows a notably higher clinical complete response rate. Because the research was conducted at a single center and given the novelty of this treatment in Iran, the number of patients included in the study was limited. Electrochemotherapy (ECT) has shown significant clinical effectiveness for superficial tumors, especially for patients who have health issues related to standard therapies or who are resistant to conventional treatments. It is generally well-tolerated, with side effects predominantly consisting of temporary pain. Ongoing research aims to expand its use in deep-seated tumors that are resistant to conventional therapies.

## Introduction

Basal cell carcinoma and squamous cell carcinoma represent 90% of head and neck cancer pathologies which accompanies with high morbidities and dysfunction of vital structures.^1,2,3^ Recurrence or advanced skin cancer in important anatomical areas is not only a therapeutic challenge for physicians but also a debilitating condition for patients. As a surgery, radiation therapy, and, in certain circumstances, chemotherapy and target therapy can cause morbidities and major deformities in these cases; electrochemotherapy can introduce the patients to lessened adverse effects.^4,5,6^

By applying local electrical pulses, pores are created in the cell membrane. Depending on the intensity and time of these pulses, the pores created are reversible or irreversible. If these pores are reversible, they can facilitate the entry of certain substances into the cell. If these holes are irreversible, the cell cannot maintain its electrochemical balance and undergoes apoptosis, which is called irreversible electroporation (IRE).

Electrochemotherapy (ECT) is a novel treatment method for unresectable cutaneous and subcutaneous tumors.^6^ It utilizes electric pulses to enhance the delivery of chemotherapy agents into cancer cells, and as a result, the efficacy of drugs will significantly increase.^7,8,9^ ECT is especially beneficial for head and neck skin cancer patients who were expected to find deformity through standard treatment modalities.^10,11^ Based on evidence, the objective response rate of electrochemotherapy was around 70%–80% in skin cancer. Therefore, high tumor control rate accompanied with minimal normal tissue damage is the main advantage of electrochemotherapy.^12^ Apart from aiding in local tumor management, electrochemotherapy (ECT) can also play a valuable role in palliative care. It has been shown to reduce pain and improve the quality of life for numerous patients suffering from advanced pancreatic ductal adenocarcinoma (PDAC).^13,14^ Electrochemotherapy therapy (ECT) is cost-effective, with an incremental cost-effectiveness ratio (ICER) of €1571.53 to achieve an additional response.^15^

With the purpose of investigating the effectiveness and adverse effects of electrochemotherapy in the most common types of skin cancers including cSCC, BCC and melanoma the present study was launched.

## Material and Methods

This study was conducted to examine the safety and effectiveness of electrochemotherapy (ECT) for treating superficial skin cancers, including basal cell carcinoma (BCC), squamous cell carcinoma (SCC), and melanoma, in an Iranian population. The main goals were to evaluate how tumors responded to ECT and to observe any side effects related to the treatment. This single-arm phase 2 clinical trial was carried out at a specialized cancer center in Tehran. A total of 26 patients with histologically confirmed skin cancer were prospectively enrolled between 2022 and 2024. All patients met the study's inclusion criteria and gave written informed consent prior to receiving treatment. The primary outcome was the tumor's clinical response one month after ECT, measured by either complete or partial reduction in tumor size. Additional outcomes included the occurrence of side effects, the need for additional treatments, and any signs of tumor recurrence during follow-up.

The study protocol was reviewed and approved by the Research Ethics Committee. The research was conducted in accordance with the ethical standards of the institutional and national research committees and with the 1964 Helsinki declaration and its later amendments. Written informed consent was obtained from all individual participants included in the study.

Patients were carefully chosen based on European Standard Operating Procedures on Electrochemotherapy (ESOPE).^16^ Inclusion and exclusion criteria by a multidisciplinary oncology group including surgeons, oncologists, pathologists, radiologists and dermatologists.

### Indications for Treatment Exclusion

- patients with pacemakers
- Patients with any previous difficulties with local anesthesia (such as lidocaine) or general anesthesia were excluded from the study.
- Intravenous injection is prohibited for patients who are allergic to bleomycin or have a history of pulmonary fibrosis. If a treatment cannot be administered as an intratumoral injection, it is not included in the study design.

### Indications for Treatment Include

- Skin metastases of any type are symptomatic due to factors such as bleeding, ulceration, exudation, odor, or pain.
- Progression of skin metastases where the symptoms mentioned above are anticipated.
- Primary skin cancers, including recurrent tumors, for which other treatment options (such as surgery, radiotherapy, and systemic therapies) have failed or are not viable.
- Patients who are receiving systemic therapy but whose skin metastases are progressing or not responding, even though there has been a satisfactory response in the internal organs.
- Patients expressing a preference for electrochemotherapy after all other treatment options have been thoroughly explained.

Then, electrochemotherapy, its advantages and disadvantages and a comparison with standard treatment was introduced to the patients. If the patient accepted, the informed consent would be obtained.

According to the ESOPE study,^16^ all nodules in a patient with multiple lesions were treated. However the biggest lesions were always pointed out. The target lesion sizes were measured and captured before and after the therapy. The ESOPE study and Standard Operating Procedure were followed in the performance of ECT.^16^

**Figure 1.**
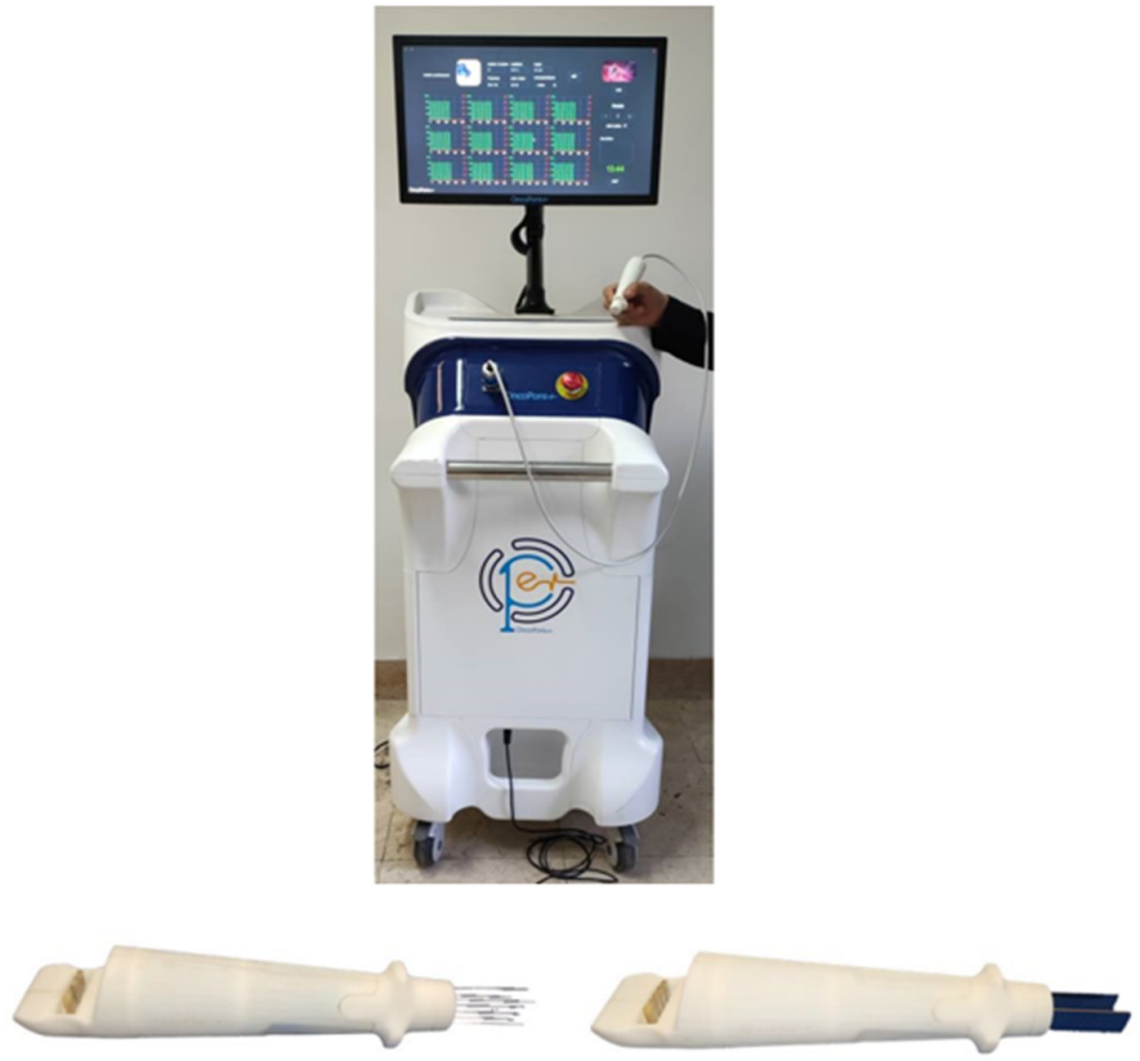
Up: Electroporation System (OncoPore, Iran). Hexagonal Needle (Bottom Left) and Double Parallel Plate (Bottom Right) Electrodes.

**Figure 2.**
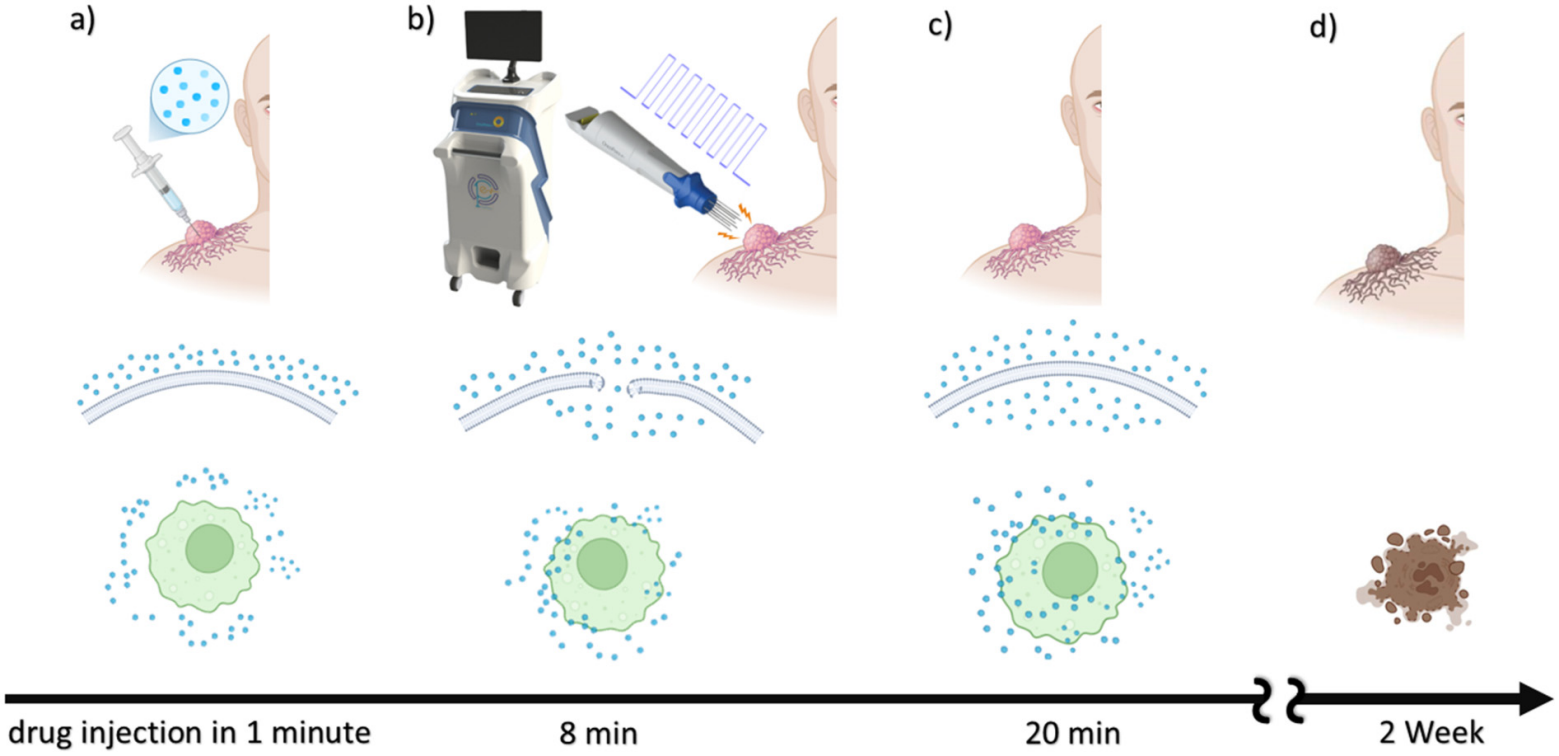
Brief Schematic of ElectroChemoTherapy. (a) Bleomycin was Administered Intravenously or Intratumoral, with a Concentration of 1000 IU/ml, Dissolved in sterile Water for 60 s (b) After 8 min, Electrical Stimulation Including 8 Monopolar Pulses with an Amplitude of 1000 V/cm is Applied (c) in About 20 min, the Entire Area of the Tumor Should be Stimulated Permeabilized the Tumor Cells for the Drug (d) After 2 Weeks, the Initial Result Will be Seen.

Bleomycin was injected intravenously (15 000 IU per body surface area (BSA dissolved in sterile water) for 60 s, 8 min before the application of electric pulses, in adherence to ESOPE guidelines. ECT procedures were performed under general anesthesia. Lesions were photographed before and after each treatment session to monitor the responses and adverse effects. The treatment was administered using the electroporation device (OncoPore, Iran) (Figure 1). Three electrode types employed for treating cutaneous and subcutaneous lesions consisted of 7 needle electrodes arranged in a hexagon shape with a central needle, two plate electrodes and parallel needle electrodes. Electric pulses were delivered at a field intensity exceeding 400 V/cm to 1000 V/cm and an amplitude exceeding 400 V, with a pulse length of 100 us. The treatment regimen included 8 electric pulses per Stimulation, generated at a frequency of 5000 Hz. Depending on the type of tumor and the electrodes used, tumors are categorized into different sections. For nodules that are smaller than one centimeter and superficial, stimulation is applied using plate electrodes placed around them. In this case, a sterile conductive gel, is utilized to ensure better contact between electrode and tumor surface. For larger or deeper tumors, needle electrodes are employed. Research indicates that hexagonal electrodes are typically used for larger tumors, while linear array electrodes offer better tumor control for tumors measuring up to 3 cm in size.^17^ In this situation, the therapeutic procedure is divided into smaller sections, each measuring one centimeter, and each section is stimulated individually. Bleomycin was injected in 1 min (bolus protocol) and 8 min before starting electrical stimulation to allow homogeneous distribution all over the tumor volume. The procedure was carried out due to ESOPE guidelines (Figure 2).

According to ESOPE^16^ nodules up to 1 cm were treated with plate electrode, nodules between 1-3 cm were treated with needle row electrodes and larger nodules were treated with hexagonal needle electrodes. Drug injection selected according to the number and size of the nodules. If the number of nodules is more than 7 or their total sizes are larger than 3 cm, the drug is injected intravenously, otherwise, the drug would be injected intratumorally. treatment strategy is summarized in Table 1.

**Table 1.** Treatment Strategy Based on Number and Size of Tumors.

	Intratumoral injection	intravenous injection	Electrode type		
			plate	parallel needle	Hexagonal needle
Tumor size	≤ 3cm	> 3cm	≤ 1cm	1 cm < size < 3cm	> 3cm
Tumor count	≤ 7	> 7			

Patients were followed for first, second, third and fourth week of treatment and in third, sixth, ninth and 12^th^ month after the treatment. At each visit, tumor clinical response and any subsequent side effects or toxicity were monitored. The diameters of target lesion were measured with calipers and ultrasonography was used to measure subcutaneous nodules. ESOPE and RECIST (Response Evaluation Criteria in Solid Tumors)^18^ criteria were used to objectively measure the lesions. Complete response (CR) was defined as disappearance of the target lesion and partial response (PR) was considered as a decrease of 30% or more in the sum of the diameters of the target lesions. Progressive disease (PD) was described as an increase of 20% or more in the sum of the diameters of the target lesions. The lesions which developed outside of the treatment field was considered as new lesions rather than recurrence.

Adverse events were evaluated using the Common Terminology Criteria for Adverse Events (CTCAE) for Skin and Subcutaneous Tissue Disorders.^19^

## Statistical Analysis

A chi-square test was used to evaluate the association between treatment response rates and cancer types (BCC, SCC, melanoma). The analysis was conducted using Python (version 3.10) with the SciPy library (version 1.11.4). Cramér's V was calculated to assess the effect size of the association.

## Results

### Patients’ Characteristics and Nodules Properties

A total of 26 patients including 17 male (65.3%) and 9 Female (34.7%) were recruited in this study, with a mean age of 64.8 years (40-83 years). Around 46% of cases were BCC and around 60% of nodules located in the head and neck. Nine patients (35%) had primary tumors without any previous treatment. Summary of nodules properties and patients’ characteristics are shown in Tables 2 and 3, respectively. The mean number of nodules treated per ECT session was 3.1. Lesion sizes varied, with a mean diameter of 2.8 cm (range 1-23 cm), and a mean of 24 electric pulse applications delivered per treatment.

**Table 2.** Nodules Site, Type, status and the Type of Electrode Used.

Site	Head and neck	15 (57.7%)	Electrode	Hexagonal	7 (26.9%)
	Trunk	1 (3.8%)		plate	7 (26.9%)
	Limb	10 (38.5%)		Parallel needle	16 (61.5%)
Type	BCC	12 (46.1%)	Status	Local (primary)	9 (34.4%)
	SCC	6 (23.1%)		Local (recurrence)	9 (34.4%)
	Melanoma	8 (30.8%)		Metastatic	8 (31.2%)

**Table 3.** patients’ characteristics.

Patient No	Gender	Age	Target lesion size*	Site	TNM (staging)	Previous Treatments	Number of ECT Sessions	Response at 1 Mo CR/PR/SD/PD	Status at last follow up	Pathology	Number of lesions	Electrode type
1	Female	57	1 × 1	Face	T1N + M0/Localized	None	2	CR	Recurrence in 6 lesions at 12^th^ month of f/u. CR at 23^th^ month	BCC, nodular type	10	plate
2	Female	52	1 × 1	Nose	T1N0M0/Localized	None	1	CR	CR at ninth month of f/u	BCC	1	plate
3	Male	75	1.5 × 1	5the finger	T2N0M0/Localized	Surgery	1	CR	CR at ninth month of f/u	BCC	1	Parallel Needle
4	Male	79	2 × 1	Face	T1N0M0/Localized	Surgery	1	CR	CR at 12^th^ month of f/u	BCC	7	plate and Parallel Needle
5	Male	68	2.5 × 2.5	Face	T4N0M0/Advanced	None	1	PR	Progression at fourth month of f/u	BCC, nodular and infiltrative	1	Parallel Needle
6	Male	73	2.5 × 2.5	Scalp	T2N0M0/Localized	None	1	CR	CR at ninth month of f/u	BCC	1	Parallel Needle
7	Male	59	2.5 × 2.5	Nose	T4N0M0/Advanced	None	1	CR	CR at ninth month of f/u	BCC	1	Parallel Needle
8	Female	76	2.5 × 2	Scalp	T2N0M0/Localized	None	1	CR	CR at 18^th^ month of f/u	BCC, superficial	1	Parallel Needle
9	Female	47	2 × 1	Nose	T4N0M0/Advanced	Surgery	1	CR	CR at seventh month of f/u	BCC, solid and infiltrative	1	Parallel Needle
10	Male	71	1 × 1.5	Nose	T2N0M0/Localized	None	1	CR	CR at seventh month of f/u	BCC	1	Parallel Needle
11	Male	67	2 × 1	Ear	T1N0M0/Localized	None	1	CR	CR at seventh month of f/u	BCC, infiltrative type	1	Parallel Needle
12	Male	65	4 × 3	Ear	T4N0M0/Advanced	Surgery Radiation therapy	1	CR	CR at 16^th^ month of f/u	BCC, basosquamous	1	Needle Hexagonal
13	Male	73	1 × 1	Lower Limb	T4N + M+	Surgery Target therapy	1	PR	Died due to stroke at second month of f/u	Melanoma	8	Plate
14	Female	40	2 × 1.5	Lower Limb	T4N + M+	Surgery Target therapy	2	PR	Progression at eighth month of f/u	Melanoma, superficial spreading type	10	Plate and Parallel needle
15	Male	81	23 × 17	Lower Limb	T4N + M+	Surgery	1	PR	Died due to brain metastasis at third month of f/u	Melanoma, nodular	2	Needle Hexagonal
16	Female	72	5.5 × 3	Upper limb	T4N + M+	Surgery	1	CR	Progression at eighth month of f/u. Died due to brain metastasis at 17^th^ month of f/u	Melanoma, acral lentiginous melanoma	2	Needle Hexagonal
17	Male	53	1.5 × 2.5	Lower limb	T1N0M0	Surgery	1	CR	CR at 18^th^ month of f/u	Melanoma, nodular type	15	Plate and Parallel Needle
18	Male	61	2 × 1.5	Lower limb	T4N + M+	Target therapy	2	PR	CR at 18^th^ month of f/u	Melanoma, nodular type	20	Plate and Parallel Needle
19	Female	58	10 × 9	Breast	T4N + M+	Surgery Target therapy Radiation therapy	1	CR	CR at seventh month of f/u	Melanoma, NA	2	Needle Hexagonal
20	Female	47	1.4 × 2	Lower limb	T4N + M+	Surgery Target therapy	1	CR	CR at 16^th^ month of f/u	Melanoma, nodular type	2	Parallel Needle
21	Female	63	3 × 3.5	Hand and forearm	T3N0M0	Surgery Radiotherapy Chemotherapy	5	CR	Recurrence in the margin at third, eighth, 11^th^, and 24^th^ month of f/u which were treated. CR at 26^th^ month of f/u	SCC, keratinizing	2	Needle Hexagonal
22	Male	47	2 × 2.5	Upper Limb	T4N + M+	Surgery radiotherapy	1	PR	Died due to lung metastasis and failure at third month of f/u	SCC, keratinizing	5	Parallel Needle
23	Male	74	2 × 1	Right ear	T1N0M0	Surgery	1	CR	CR at ninth month of f/u	SCC	2	Parallel Needle
24	Male	69	3.5 × 1	Right ear	T4N0M0	Surgery	3	CR	Recurrence at sixth and eighth month of f/u. CR at 11^th^ month of f/u	SCC, keratinizing	2	Needle Hexagonal
25	Male	75	7.5 × 6	Face	T4N0M0	None	1	PR	Progression at third month of f/u	SCC	1	Needle Hexagonal
26	Male	83	1 × 2	Face	T4N0M0	Surgery	2	PR	CR at 14^th^ month of f/u (after second ECT)	SCC	4	Parallel Needle

*In the case of multiple lesions, the biggest lesions were always pointed out.

CR: complete response, PR: partial response, f/u: follow up, TNM: the extent of the tumor (T), extent of spread to the lymph nodes (N), and presence of metastasis (M).

### Tumor Response

As it was shown in Table 4, more tumor complete response during first month of treatment was seen in BCC nodules and most of partial responses were seen in melanoma nodules (Chi-square test, p-value 0.001).

**Table 4.** Response Rate in Different Types of Nodules.

BCC	26	1
SCC	6	10
Melanoma	21	40
	CR	PR

Most of the patients (20/26) had received one session of ECT. ECT was repeated in 3 patients due to partial response and in 3 patients because of recurrence after the first ECT session. Of the three patients who underwent second session of electrochemotherapy due to partial response, two achieved complete response (case NO. 18 and 26), and one progressed (case NO. 14). Recurrence was recorded in three patients; in case NO. 1 new lesions both inside and outside of the field of electrochemotherapy was happened after 12 months. In case NO. 21, recurrences were happened in the margin of previous treated tumor 4 times after first treatment. In case NO. 21, recurrences occurred in the margin of previous treated tumor 2 times after the first ECT.

### Adverse Effects

ECT sessions were well-tolerated by most patients, and no major complications have occurred.

A significant majority of symptoms (86.5%) resolved within the first 48 h after the procedure.^20^ An acute effect occurs within the first week following treatment. A chronic effect persists for more than five weeks. A subacute effect has a duration longer than an acute effect but does not last as long as a chronic effect. The skin reaction can be compared to earlier studies that reported a 44.6% occurrence of skin ulcers.^20^ Pain (24.2-92.0%) and erythema (16.6-42.0%) were the most frequent toxicities.^21^ After 12 weeks, there have been no reports of pain or any other complications associated with the electrochemotherapy treatment and only the effectiveness of the treatment is evaluated.

Table 5 summarizes the adverse effect in different time intervals.

**Table 5.** Adverse Effects.

	grade	immediate	acute (first week)	Subacute (1-4 week)	Chronic (5-12 weeks)
Pain of the skin	grade 1: mild pain	12	16	17	17
	grade 2: moderate pain	10	9	8	8
	grade 3: severe pain	4	1	1	1
Skin ulceration	grade 1	0	1	6	7
	grade 2	0	3	8	11
	grade 3	0	2	5	7
	grade 4	0	0	1	1
Skin hyperpigmentation	grade 1	0	0	1	3
	grade 2	0	0	0	0
Skin hypopigmentation	grade 1	0	0	8	13
	grade 2	0	0	0	0

### Survival Data

The mean overall and progression free survival for all patients in this study was 11.4 and 9.5 months respectively. During the study, four patients died; three of them had suffering from metastatic melanoma and one of them was metastatic cSCC.

#### Overview of Some Cases

**Figure 3.**
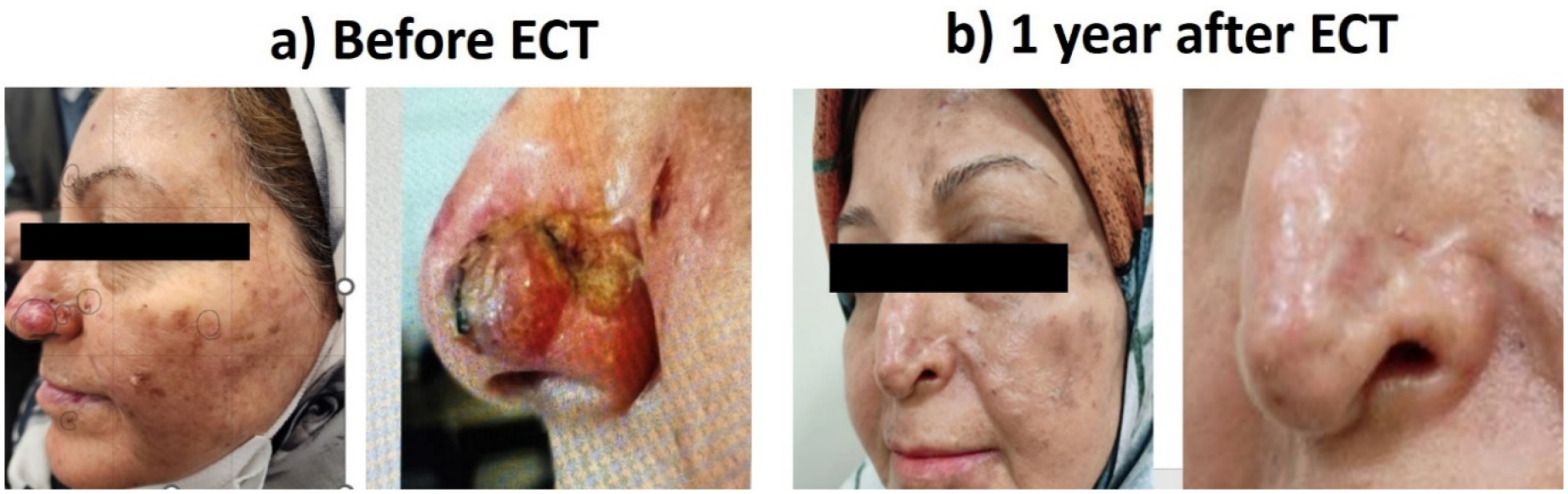
BCC Nodules.

Case 1: A 55-years-old lady who was suffered from Xeroderma Pigmentosa with multiple BCC nodules on the nasal alar and face (Figure 3).

**Figure 4.**
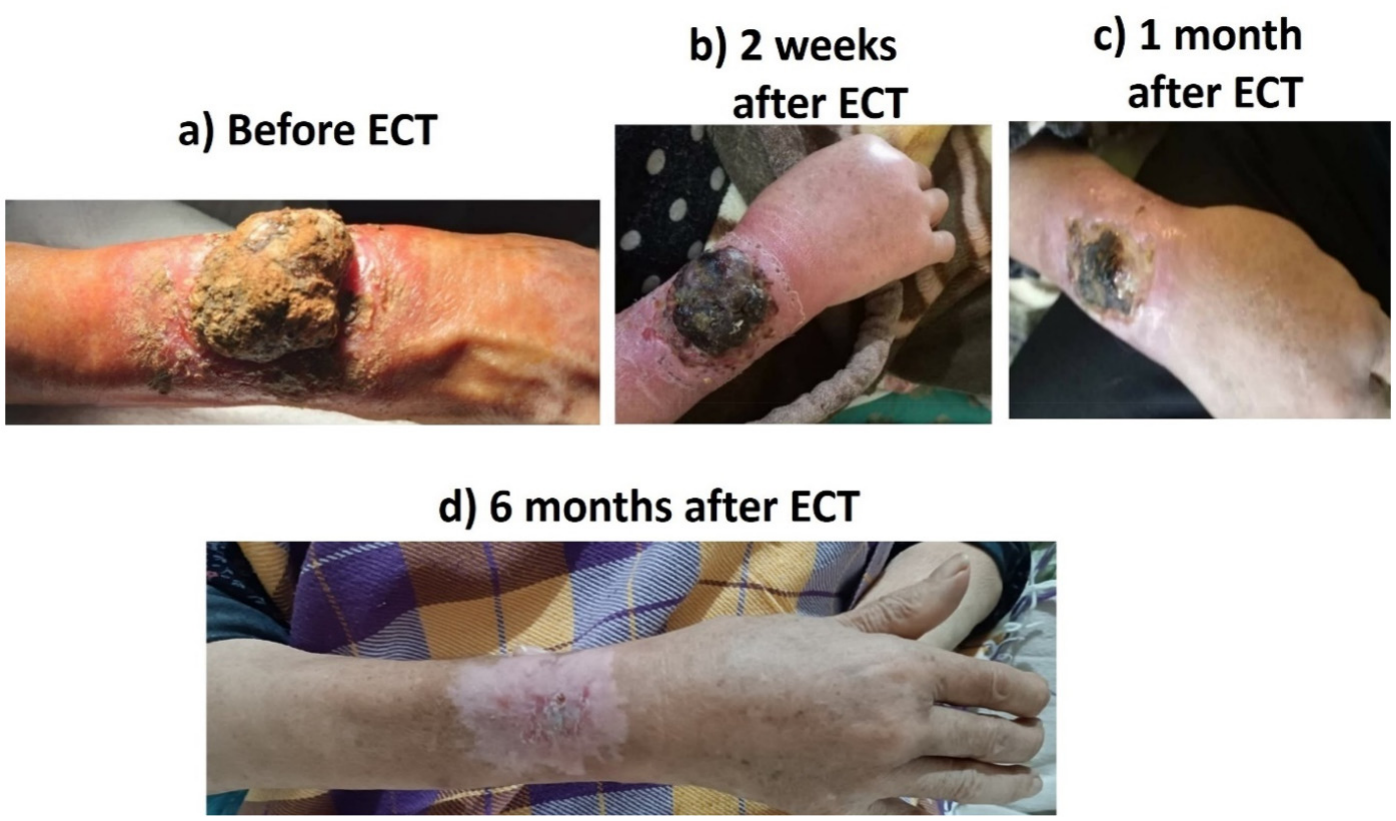
Melanoma.

Case 2: A 72-year-old lady with metastatic melanoma of the right forearm with axillary involvement (stage IIIB) who did not accept limb amputation. Axillary lymph nodes were dissected and meanwhile, the tumor was treated by ECT (Figure 4).

**Figure 5.**
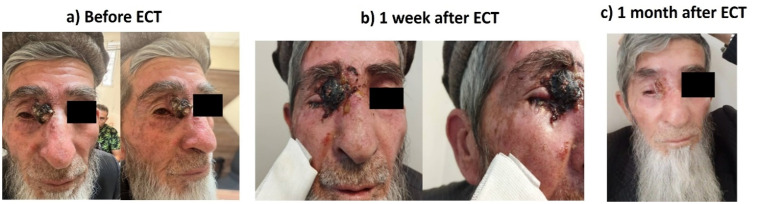
BCC Lesion.

Case 3: He is an advanced case of BCC which involves orbital muscles and eyelid. Electrochemotherapy was applied to sparing his vision rather than eye depletion (Figure 5).

**Figure 6.**
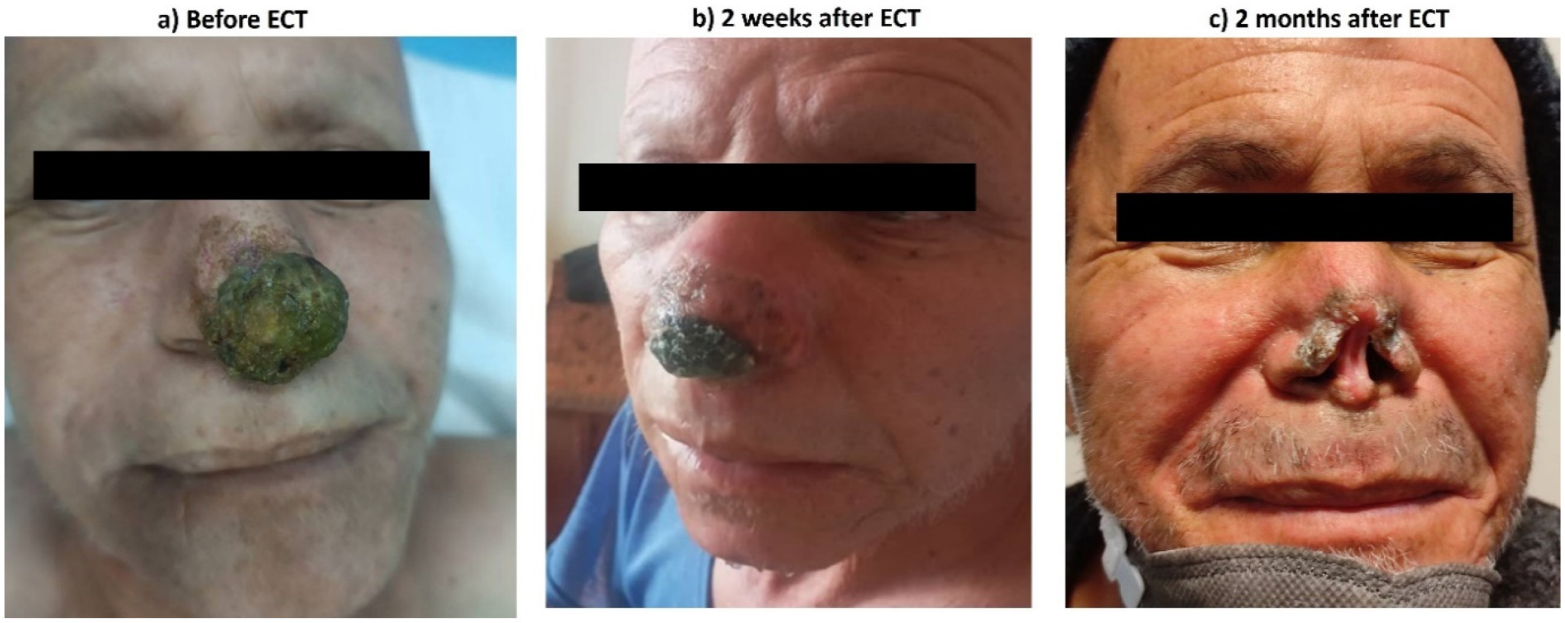
SCC Lesion.

Case 4: A 70-year-old patient who was suffered from metastatic lung SCC and SCC in nose, ECT was done by local nerve blocking (Figure 6).

**Figure 7.**
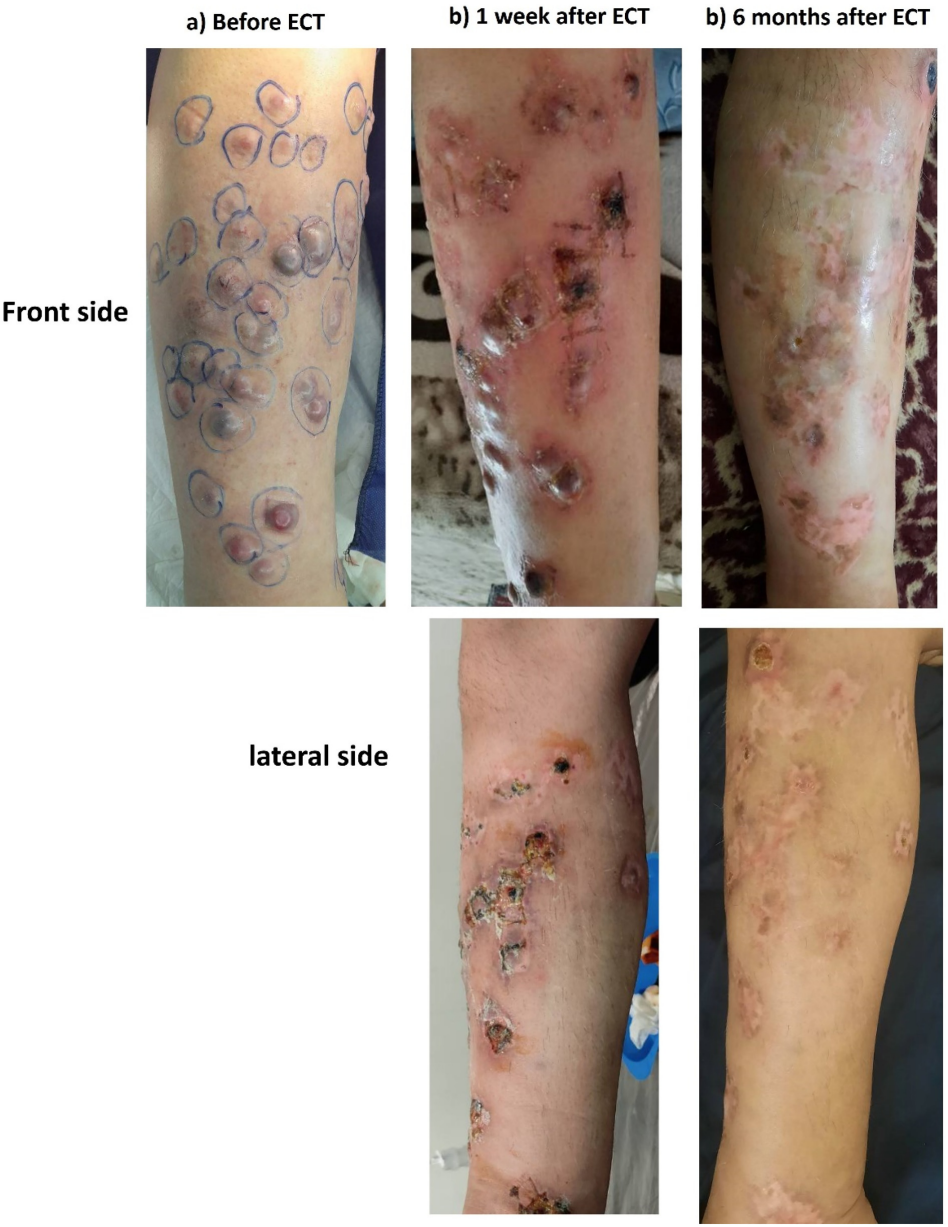
Metastatic Melanoma.

Case 5: A 45-year-old man who had progressed during treatment with anti-PDL1 (pembrolizumab). He was extraordinary controlled by 2 electrochemotherapy sessions which was lasted about 1 year (Figure 7).

## Discussion

The safety and effectiveness of electrochemotherapy in the management of skin tumors, such as metastatic melanoma, cutaneous squamous cell carcinoma (cSCC), and basal cell carcinoma (BCC), have been shown in this prospective trial. In the present study, the overall response rate at the first month of treatment was 100% (consist of 51% and 49% complete and partial response respectively). These findings align with existing literature that highlights the effectiveness of ECT to obtain 50% complete response after 60 days.^22^ Based on a meta-analysis, which includes 29 studies and 1503 patients with skin cancer or metastasis to the skin from other origins, complete response and objective response were reported in 47% and 82% of lesions respectively.^23,24^ BCC showed the highest complete response rate among these three groups. We showed that 96% of BCCs nodules achieved complete response within two months of treatment. Similarly, a recent systematic review of 45 patients had shown a 92% complete response in BCCs after two months of treatment, suggesting that increasing the number of patients would lead to a decrease in CR.^25^ The same results were observed in a separate study using a standard dose of bleomycin. The complete tumor response at 2 months after electrochemotherapy with the reduced bleomycin dose was 100%, while the response with the standard bleomycin dose was 96%.^26^

Furthermore, cSSC nodules reached 37.5% complete response after the first session of ECT. Complete response rate in cSCC was reported between 12% to 66% in different case series which was corelated to the tumor size and the setting (primary vs recurrence) in many studies^6,27^ . Similarly, response rate in malignant melanoma nodules was comparable with previous studies^21,28^ .Although ESOPE study reported a higher complete response rate of 74%, it included the smaller lesions size in their cohort.^29^ In the ESOPE classification, tumors and electrode types are categorized based on tumor diameters of 0.8 cm and 2 cm. However, a recent study has indicated that parallel needles can be more effective for tumors up to 3 cm in size.^17^ As a result, we have revised the classifications to reflect diameters of 1 cm and 3 cm. Another multi-institutional study reported lower effectiveness of ECT on larger tumors with a maximal diameter of 3 cm or more.^22^

It is worth noting that ECT was helpful for older patients or cases with other comorbidities. Current treatment strategies are not always applicable to these patients. ECT was an effective treatment approach that was accepted by patients, provided a good clinical response, with few adverse effects, and improved quality of life. After one week of treatment, the pain caused by the stimulations disappeared. Patients who had experienced a decline in their quality of life due to pain from their condition were relieved by the reduction of pain in the treated areas. One patient, who had a widespread melanoma tumor and was unable to walk because of the severe pain, experienced a significant reduction in pain after ECT treatment and was able to return to his normal life. It was feasible to repeat ECT as it was well-tolerated, which increased the possibility of obtaining the complete clinical response.

Despite the promising results, this study has limitations such as the small sample size. Future research should be designed to focus on larger and randomized controlled trials to validate these findings.

## Conclusion

Our experience showed that electrochemotherapy (ECT) could be apllied as a well telorated less radical alternative in skin cancer recurrences (SCC & BCC) and in transit metastasis (melanoma IIIB and C) to extensive re-surgery, especially for patients with Head and Neck cancers who wouldn’t accept the surgery due to the cosmetic effects. Managing the bleeding and differing the up staging of metastatic melanoma from ulcer state to bleeding state with lowest side effects were another observed benefit of ECT.

## Abbreviations

ECT: ElectroChemoTherapy
BCC: Basal Cell Carcinoma
cSCC: cutaneous Squamous Cell Carcinoma
NMSC: Non-Melanoma Skin Cancers
MAPK: Mitogen-Activated Protein Kinases
HPV: Human PapillomaVirus
NICE: National Institute for Health and Care Excellence
ESMO: European Society for Medical Oncology
CTCAE: Common Terminology Criteria for Adverse Events
ESOPE: European Standard Operating Procedures on Electrochemotherapy
CR: Complete Response
PR: Partial Response
SD: Stable Disease
PD: Progressive Disease
